# Comparison between gaits after a medial pivot and posterior stabilized primary total knee arthroplasty: a systematic review of the literature

**DOI:** 10.1186/s42836-023-00165-8

**Published:** 2023-03-17

**Authors:** Salvatore Risitano, Giorgio Cacciola, Marcello Capella, Francesco Bosco, Fortunato Giustra, Federico Fusini, Pier Francesco Indelli, Alessandro Massé, Luigi Sabatini

**Affiliations:** 1 Department of Orthopaedic Surgery and Traumatology, University of Turin, 10126 Turin, Italy; 2 Department of Orthopaedic Surgery and Traumatology, CTO Hospital of Turin, Città Della Salute E Della Scienza, 10126 Turin, Italy; 3 Department of Orthopaedics and Traumatology, Ospedale San Giovanni Bosco—ASL Città di Torino, Piazza del Donatore Di Sangue, 3, 10154 Turin, Italy; 4 Department of Orthopaedic Surgery, Regina Montis Regalis Hospital, 12084 MondovìCuneo, Italy; 5 Department of Orthopaedic Surgery and Bioengineering, Stanford University School of Medicine, Palo Alto Veterans Affairs Health Care System (PAVAHCS), Palo Alto, CA 94304 USA

**Keywords:** Total knee replacement, Posterior stabilised, Medial pivot, Kinematics, Walking, Gait analysis

## Abstract

**Background:**

Total knee arthroplasty (TKA) is one of the most performed orthopedic procedures worldwide. While excellent efficacy has been reported, about 20% of patients are not satisfied with the result. A potential cause is the problematic reproduction of knee kinematics. This systematic review examines gait analysis studies in primary medial pivot (MP) and posterior stabilized (PS) TKAs to investigate the differences between the two prosthesis designs.

**Methods:**

A systematic review was conducted by following PRISMA guidelines. Five databases (PubMed, Medline, Embase, Scopus and the Cochrane Database of Systematic Reviews) were analyzed, and eligible articles were evaluated in terms of the levels of evidence. The methodological quality of the articles was assessed by using the MINORS scoring. This review was registered in PROSPERO.

**Results:**

Nine studies were included. Gait analysis was performed in 197 MP TKA and 192 PS TKA patients. PS TKA cases showed (*P* < 0.05) a significantly higher peak of knee flexion angle during the swing phase, greater knee flexion angle at toe-off, an increased knee adduction angle, higher knee flexion and extension moment, increased anterior femoral roll during knee flexion and anterior translation on medial and lateral condyle during knee flexion compared to MP TKA. MP TKA showed statistically significant (*P* < 0.05) higher knee rotational moment and greater tibiofemoral external rotation motion during knee flexion than PS TKA. No statistically significant difference (*P* > 0.05) was reported regarding gait spatial–temporal parameters. The Forgotten Joint Score (FJS) and Western Ontario and McMaster Universities Comparison in terms of Arthritis Index (WOMAC) score (mean stiffness) showed that MP TKA yielded significantly better results than PS TKA.

**Conclusions:**

This systematic review revealed significant kinematic and kinetic differences between MP and PS TKA at all gait analysis phases. Furthermore, the considerable difference between TKA design and the kinematics of healthy knee were highlighted in this study.

**Level of evidence:**

III.

## Background

Total knee arthroplasty (TKA) is a reliable and cost-effective surgical procedure for treating symptomatic end-stage knee osteoarthritis [1]. Currently, more than 500,000 TKAs are performed annually in the United States, with a projected increase of 670% by 2030 [2, 3]. De Steiger et al*.*, in their analysis of national registers, have reported an excellent long-term survival of implants, with a revision rate of 5.2% after ten years and a rate of 7.3% after 15 years [4].

Despite the low reoperation rate, approximately 20% of patients remain dissatisfied after TKA, and this percentage has remained unchanged over the past decades despite advances in surgical techniques and implant design [5–7]. The failure to reproduce the physiological knee kinematics after TKA is often reported as a major cause of patient dissatisfaction [8, 9]. In recent years, various prosthetic designs have been developed to improve clinical outcomes and patient satisfaction: many of those designs were presented by manufacturers with a special characteristic of reproducing more natural knee kinematics [10, 11]. According to the latest report by the Australian Arthroplasty Registry, implants characterized by lower levels of constraints (cruciate or bicruciate retaining) are currently most frequently used in TKAs. The use of posterior stabilized (PS) designs is decreasing while the use of medial congruent TKAs is steadily increasing worldwide [4].

Insall and Burstein first introduced a PS TKA model to overcome limitations in the range of motion (ROM) and the anterior femoral sliding from knee flexion to extension, typical of total condylar knee implants [12]. The most important feature of the PS TKA design was the post-cam mechanism, providing a constraint to limit the anterior translation of the femur through the tibia (“paradoxical motion”), ensuring femoral rollback with progressive knee flexion [12, 13]. The PS TKA design has the theoretical advantage of allowing for easier balancing of severe coronal deformities, reducing polyethylene wear, and improving maximum flexion compared to total condylar knee prosthesis. Nevertheless, PS TKAs have potential drawbacks, including increased tibial liner wear or breakage, an increase in the rate of postoperative anterior knee pain, and the additional bone resection necessary to accommodate the femoral box [14, 15].

Medial pivot (MP) TKA designs aim to reproduce the natural knee kinematics, where the medial femoral condyle is congruent on the concave medial tibial plateau, acting as a ball and socket mechanism. In contrast, on a flat tibial surface, the lateral femoral condyle shifts anteriorly first and posteriorly later during knee extension and flexion movements [10, 11]. Developed through Freeman and Pisnkerova's kinematic studies [16, 17] and first implanted in 1994, MP designs tend to achieve more physiological knee kinematics, better coronal and sagittal stability, and reduced polyethylene wear [18–23].

This systematic review examines comparative gait analysis studies in the primary medial pivot and posterior stabilized TKA to investigate the kinematic and kinetic differences between these two prosthetic designs on (1) the sagittal plane, (2) the coronal plane, (3) in relative tibiofemoral motion, (4) in the spatial–temporal parameters, and finally in the (5) clinical scores.

## Materials and methods

This systematic review of the literature was performed in accordance with the Preferred Reporting Items for systematic reviews and meta-analyses (PRISMA) guidelines [20, 24–26]. The literature search was conducted by three independent reviewers (G.C., F.B., and F.G.) to search for comparative gait analyses of MP *vs*. PS TKA studies. In case of discrepancies, a fourth author (LS) was involved to resolve any doubts or disagreements.

### Search strategy and study screening

**Fig. 1:**
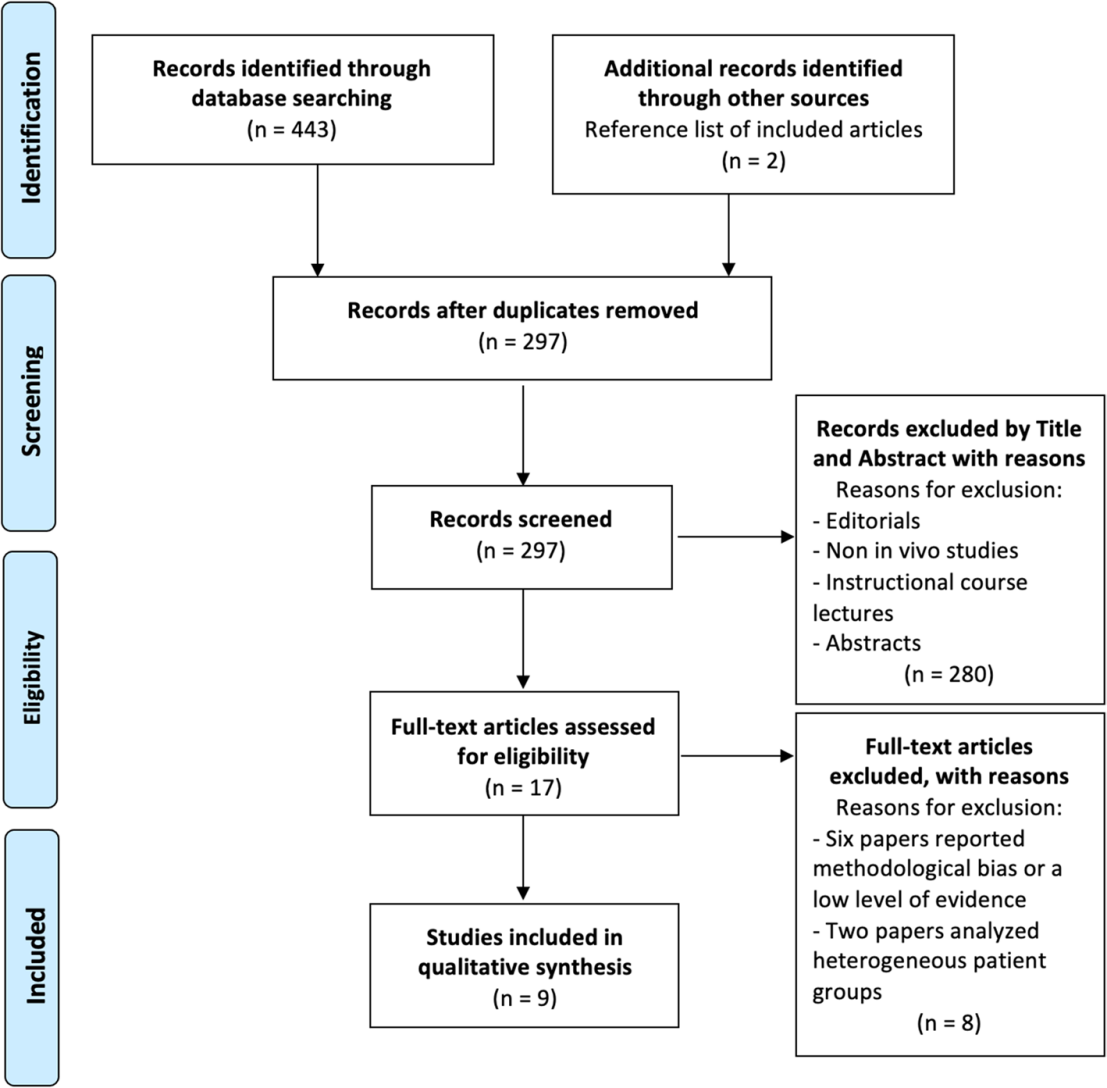
Preferred reporting items for systematic review and meta-analysis (PRISMA) flow diagram of studies included in the systematic review

The literature search was conducted in the US National Library of Medicine (PubMed/Medline), Embase, Scopus and the Cochrane Database of Systematic Reviews by using the following MeSH terms without limitation up to August 2022: “medial pivot”, “medial congruent”, “posterior stabilised”, “MP”, “PS”, “kinetic”, “kinematic”, “fluoroscopy”, “gait analysis”, “in vivo”, “knee arthroplasty”. With the above MeSH terms, the initial search produced 433 studies. After removal of duplicates, 297 studies remained for analysis. After title and abstract analysis, the full text of 17 potentially included studies was assessed for eligibility against inclusion and exclusion criteria. Nine studies that directly compared the results of gait analysis of MP *vs*. PS TKA were included in the final analysis [27–35]. Supplementary relevant articles were searched through the reference list of included studies. The PRISMA flow chart to report the study selection is shown in Fig. 1.

### Inclusion and exclusion criteria

Inclusion criteria for the reviewed studies were articles published until September 2022, with full text available, written in English, that reported knee kinematic data in the frontal or sagittal planes or relative tibiofemoral movement or gait parameters, RCTs, prospective and retrospective studies with Oxford Centre for Evidence-Based Medicine 2011 Levels of Evidence (LoE) 1 to 4 [36]. Non-comparative studies, non-in vivo studies, editorials, instructional course lectures or abstracts for international meetings were excluded from the search. We also excluded studies with LoE 5 for quality control.

### Evaluation of methodological quality

The level of evidence analysis was determined by using the Oxford Centre for Evidence-Based Medicine Levels of Evidence [36]. Additional assessment of the studies’ quality was conducted by three authors (G.C., F.B., and F.G.) according to the Methodological Index for Non-randomised Studies (MINORS) criteria [37]. A fourth author (L.S.) resolved any cases of disagreement. All authors participated equally in the study design, manuscript preparation, and final review. This systematic review was registered on the International Prospective Register of Systematic Reviews (PROSPERO), CRD42022343517, in July 2022 [38].

### Data extraction

Two reviewers (G.C. and F.B.) collected data from the selected studies and inserted them into a standard template. Initially, demographic data such as age, relevant surgical information, gait analysis protocol, and a minimum follow-up of the studies were extracted. Then, information regarding the kinematic and kinetic parameters, the tibiofemoral movement, the gait spatial–temporal parameters, and the clinical scores was collected.

### Statistical analysis

Statistical analysis was performed by employing R software, version 4.0.5 (2020; R Core Team, Vienna, Austria). Descriptive statistical analysis was conducted for all data extracted from the included studies. Mean values with a measure of variability as standard deviation (SD) or range (minimum–maximum) were calculated for continuous variables. Absolute number and frequency distribution were calculated for categorical variables. Furthermore, when available, the *P* values of the variable analysis comparisons of the differences included in the various studies were reported.

## Results

### Study characteristics

Gait analysis was performed in 197 MP TKA (average age, 69.4 ± 3.9 years) and in 192 PS TKA patients (average age, 68.6 ± 4.4). Three studies [23, 26, 27] performed gait analysis also in a control group involving 40 healthy knees (average age, 36.1 ± 5.7). The average study quality based on MINORS criteria was 14.6 ± 2.6. The main demographic characteristics are reported in Table 1.

**Table 1:** Main demographic characteristics of patients recruited in studies included in the systematic review

**Author and Publication Year**	**LoE**	**MINORS Score**	**MP TKA**			**PS TKA**			**Healthy Knees**		**Gait Analysis Protocol**	**min FU**
			TKA design	*n*	Age (Mean value ± SD/Range) (y.o.)	TKA design	*n*	Age (Mean value ± SD/Range) (y.o.)	*n*	Age (Mean value ± SD/Range) (y.o.)		
Stolarczyk et al*.*,2022 [27]	III	20	K-Mod (Gruppo Bioimpianti)	28	68 ± 6.5	NexGen (Zimmer)	28	71 ± 5	20	59.4 ± 7.9	Three-dimensional gait analysis with BTS SMART device (BTS Bioengineering, Quincy, MA, USA)	2 years
Ghirardelli et al*.*, 2021 [28]	III	14	Persona MC (Zimmer)	6	63.8 ± 9.2	Persona PS (Zimmer)	6	59.4 ± 7.9	/	/	3D kinematic analysis using a multicamera optoelectronic system (Qualisys AB, Gothenburg, Sweden)	9 months
Tan et al*.*, 2021 [29]	III	16	MP (Evolution, MicroPort Orthopedics)	12	70.8 ± 3.9	Genesis II (Smith & Nephew)	12	67.7 ± 4.9	/	/	Walk on a treadmill under a dual fluoroscopic imaging system (BV Pulsera, Philips, Andover, MA, USA)	1 year
Bianchi et al*.*, 2020 [30]	III	12	MP (Evolution, MicroPort Orthopedics)	16	72 (68–76)	Persona PS (Zimmer)	16	71 (69–74)	/	/	Treadmill walk with a video-recording device (Walker View 3.0, Tecnobody, Dalmine, Italy)	1 year
Miura et al*.*, 2020 [31]	II	16	MP (Evolution, MicroPort Orthopedics)	20	72.6 ± 5.5	Persona PS (Zimmer)	20	73.1 ± 5.5	10	27.8 ± 4.7	3D motion analysis system consisted of 8 infrared cameras (ProReflex, Qualisys AB Inc., Gothenburg, Sweden) and 2 force plates (OR6; Advanced % Technology Inc., Watertown, MA, USA)	1 year
Esposito et al*.*, 2020 [32]	III	12	GMK Sphere (Medacta)	20	73.3 ± 3.5	Persona PS (Zimmer)	20	70.5 ± 4.7	20	32.1 ± 6.7	Motion tracking with eight infrared cameras (SMART-DX 700, BTS Bioengineering, Milan, Italy)	1 year
Gray et al*.*, 2020 [33]	II	14	GMK Sphere (Medacta)	26	67.3 ± 6.4	GMK PS (Medacta)	23	66.8 ± 7.3	/	/	3D motion data were recorded using a 9-camera motion capture system (VICON Motion Systems, Ltd, Oxford, UK) Ground reaction forces were measured using two portable strain-gauged force plates (AMTI Accugait, Watertown, MA, USA)	6 months
Benjamin et al*.*, 2018 [34]	II	15	Saiph Knee (MatOrtho)	45	62.4 (54–71)	Triathlon (Stryker)	45	64.8 (58–73)	/	/	Instrumented treadmill with force plates (Kistler Gaitway, Kistler Instrument Corporation, Amherst, NY, USA)	1 year
Papagiannis et al*.*, 2016 [35]	III	12	MP fixed-bearing prosthesis (Advance, Wright Medical)	24	70.3 ± 1.9	/	22	72.9 ± 1.5	/	/	Eight optoelectronic cameras (BTS) integrated with two force plates (Kistler Gaitway, Kistler Instrument Corporation, Amherst, NY, USA)	1 year

*MP* Medial pivot, *PS* Posterior stabilised, *TKA* Total knee arthroplasty, *LoE* levels of evidence, *MINORS score* Methodological index for non-randomized studies score, *N* Number of evaluation cases, *SD* Standard deviation, *y.o.* years old, *Min* Minimum, *FU* Follow-up, *MC* Medial congruent, *FU* Follow-up; /: not reported

### Kinematic parameters

Six studies reported kinematic data during gait analysis (Table 2) [28, 29, 31–33, 35]; two studies [29, 32] reported the peak of knee flexion angle during the swing phase; one of them [32] reported a statistically significantly greater peak in PS TKA compared to MP TKA. Two studies [28, 32] reported knee flexion angle at heel strike with no statistical difference found between the groups. No statistical differences were reported in knee flexion angle at the early stance phase [31], midstance knee flexion angle [28], knee flexion ROM [35], and knee abduction angle [33]. One study reported a significantly reduced knee flexion angle at toe-off in MP compared to PS TKA [32], and another reported a significantly greater knee adduction angle with PS than with MP TKA [28].

**Table 2:** Kinematic parameters

Kinematic Parameters	Author and Publication Year	MP TKA	PS TKA	*P* Value
		**Mean Value ± SD/Range**	**Mean Value ± SD/Range**	
Peak of Knee Flexion Angle During the Swing Phase	Tan et al*.*, 2021 [29]	52.4° ± 7.4°	50.1° ± 3.6°	> 0.05
	Esposito et al., 2020 [32]	47.3° ± 7°	55.6° ± 8.4°	< 0.05
Knee Flexion Angle at Heel Strike	Ghirardelli et al., 2021 [28]	3.1° ± 1.3°	7.9° ± 5.3°	> 0.05
	Esposito et al., 2020 [32]	2.2° ± 5.1°	5° ± 6.9°	> 0.05
Knee Flexion Angle at The Early Stance Phase	Miura et al., 2020 [31]	9.6°	12.1°	> 0.05
Knee Flexion Angle at Toe-Off	Esposito et al., 2020 [32]	24.2° ± 5.7°	30.2° ± 8°	< 0.05
Midstance Knee Flexion Angle	Ghirardelli et al., 2021 [28]	14° ± 4.3°	19.2° ± 5.4°	> 0.05
Knee Flexion ROM	Papagiannis in et al., 2016 [35]	117.9° ± 3.1°	117.9° ± 3.2°	> 0.05
Knee Adduction Angle	Ghirardelli et al., 2021 [28]	4.7° ± 1.4°	7.9° ± 2.2°	< 0.05
Knee Abduction Angle	Gray et al., 2020 [33]	0.2°	0°	> 0.05

*MP* Medial pivot, *PS* Posterior stabilised, *SD* Standard deviation, *°* Degree, *ROM* Range of motion

### Kinetic parameters

Three studies reported kinetic parameters during gait analysis (Table 3) [28, 32, 35]; two studies reported the knee adduction moment [28, 32], and one reported the knee abduction moment [32], but no statistical differences in the two parameters between the two groups were reported. Two studies reported knee flexion moment [28, 32], and in both studies, patients receiving MP TKA showed a statistically significantly higher moment than their counterparts receiving PS TKA. A significantly higher value was reported for PS TKA regarding knee extension moment [28] and for MP TKA regarding knee rotation moment [28]. No statistical differences were reported in the peak internal rotation moment between the two groups [35].

**Table 3:** Kinetic parameters

**Kinetics Parameters**	**Author and Publication Year**	**MP TKA**	**PS TKA**	***P* Value**
		**Mean Value ± SD/Range**	**Mean Value ± SD/Range**	
Knee Adduction Moment	Ghirardelli et al., 2021 [28]	2.21 ± 0.64%BW*Ht	2.09 ± 0.31%BW*Ht	> 0.05
	Esposito et al., 2020 [32]	-0.04 ± 0.02 Nm/kg	-0.06 ± 0.06 Nm/kg	> 0.05
Knee Abduction Moment	Esposito et al., 2020 [32]	0.63 ± 0.14 Nm/kg	0.65 ± 0.12 Nm/kg	> 0.05
Knee Flexion Moment	Ghirardelli et al., 2021 [28]	2.34 ± 1.16%BW*Ht	3.78 ± 1.42%BW*Ht	** < 0.05**
	Esposito et al., 2020 [32]	-0.34 ± 0.09 Nm/kg	-0.48 ± 0.16 Nm/kg	** < 0.05**
Knee Extension Moment	Esposito et al., 2020 [32]	0.21 ± 0.12 Nm/kg	0.27 ± 0.28 Nm/kg	** < 0.05**
Peak Internal Rotation Moment	Papagiannis et al., 2016 [35]	0.24 ± 0.04 Nm/kg	0.23 ± 0.06 Nm/kg	> 0.05
Knee Rotational Moment	Ghirardelli et al., 2021 [28]	0.88 ± 0.14%BW*Ht	0.64 ± 0.09%BW*Ht	** < 0.05**

*MP* Medial pivot, *PS* Posterior stabilised, *SD* Standard deviation, *°* Degree, %BW*Ht % percentage, *BW* Body weight, *Ht* Height, *Nm/Kg* Newton × Meter/Kilogram

### Tibiofemoral movement

Four studies reported the tibiofemoral movement during gait analysis (Table 4) [29, 31, 33, 35]. Three studies [29, 31, 33] reported the anterior femoral roll during knee flexion; one of them [33] reported that PS TKA showed a significantly greater anterior femoral roll than MP TKA. One study reported the posterior translation on the medial condyle during knee flexion [29] with no statistical difference revealed between the two groups. One study reported a statistically significantly greater anterior translation on the medial condyle during knee flexion in the PS TKA group [33]. Two studies [29, 33] reported the anterior translation on lateral condyle during knee flexion, and one study [33] reported a significantly greater anterior translation in the PS TKA group. One study [33] reported the lateral translation on lateral and medial condyle during knee flexion, but no difference between the groups was reported. Three studies [29, 31, 33] reported the tibiofemoral external rotation motion during knee flexion, and only one of them [33] reported a statistically significant increase in the MP TKA group. Lastly, one study [35] reported the overall relative tibiofemoral internal/external rotation during the gait cycle, but no differences were noted between the two groups.

**Table 4:** Tibiofemoral movement

**Tibiofemoral Movement**	**Author and Publication Year**	**MP TKA**	**PS TKA**	***P* Value**
		**Mean Value ± SD/Range**	**Mean Value ± SD/Range**	
Anterior Femoral Roll During Knee Flexion	Tan et al., 2021 [29]	4.5 ± 2.3 mm	6.6 ± 2.7 mm	> 0.05
	Miura et al., 2020 [31]	8 mm	7.3 mm	> 0.05
	Gray et al., 2020 [33]	4.4 mm	9.9 mm	** < 0.05**
Posterior Translation on Medial Condyle During Knee Flexion	Tan et al., 2021 [29]	3.9 ± 5.5 mm	3.6 ± 6 mm	> 0.05
Anterior Translation on Medial Condyle During Knee Flexion	Gray et al., 2020 [33]	3 mm	11.1 mm	** < 0.05**
Anterior Translation on Lateral Condyle During Knee Flexion	Tan et al., 2021 [29]	8.9 ± 9.2 mm	4 ± 4.7 mm	> 0.05
	Gray et al., 2020 [33]	7.3 mm	10.5 mm	** < 0.05**
Lateral Translation on Medial Condyle During Knee Flexion	Gray et al., 2020 [33]	1.7 mm	2.3 mm	> 0.05
Lateral Translation on Lateral Condyle During Knee Flexion	Gray et al., 2020 [33]	1.8 mm	2.2 mm	> 0.05
Tibiofemoral External Rotation Motion During Knee Flexion	Tan et al., 2021 [29]	5.9° ± 4.8°	6.2° ± 4.1°	> 0.05
	Miura et al., 2020 [31]	4.7°	4.1°	> 0.05
	Gray et al., 2020 [33]	6.2°	2.7°	** < 0.05**
Subtracting The Maximum Internal to The Maximum External Rotation Angle During Knee Movement	Papagiannis et al., 2016 [35]	21.6° ± 5.8°	19.7° ± 7.4°	> 0.05

*MP* Medial pivot, *PS* Posterior stabilised, *SD* Standard deviation, *mm* Millimetres, *°* Degree

### Gait spatial–temporal parameters

Six studies reported gait spatial–temporal parameters (Table 5) [27, 28, 30, 32–34]. Five studies [28, 30, 32–34] reported the walking speed. Three studies [27, 30, 34] reported the cadence. Three studies [27, 30, 34] reported the step length. One study reported the stride length [32]. Two studies reported the stance time [30, 34]. Lastly, one study reported the base of support [32]. In none of these gait spatial–temporal parameters were stastistically significant differences reported between MP and PS TKA.

**Table 5:** Gait spatial–temporal parameters

**Gait Spatial–Temporal Parameters**	**Author and Publication Year**	**MP TKA**	**PS TKA**	***P* Value**
		**Mean Value ± SD/Range**	**Mean Value ± SD/Range**	
Walking Speed	Bianchi et al., 2020 [30]	1.24 m/s	1.00 m/s	> 0.05
	Esposito et al., 2020 [32]	0.94 ± 0.19 m/s	1.02 ± 0.12 m/s	> 0.05
	Gray et al., 2020 [33]	0.87 ± 0.14 m/s	0.86 ± 0.14 m/s	> 0.05
	Ghirardelli et al., 2021 [28]	1.25 m/s	1.30 m/s	> 0.05
	Benjamin et al., 2018 [34]	1.16 m/s	1.21 m/s	> 0.05
Cadence	Bianchi et al., 2020 [30]	0.68 (0.61–0.76) m	0.62 (0.57–0.66) m	> 0.05
	Stolarczyk et al., 2022 [27]	0.62 ± 0.24 m	0.70 ± 0.23 m	> 0.05
	Benjamin et al., 2018 [34]	104 steps/min	105.86 steps/min	> 0.05
Step Length	Bianchi et al., 2020 [30]	0.25 (0.21–0.32) m	0.21 (0.19–0.23) m	> 0.05
	Stolarczyk et al., 2022 [27]	0.43 ± 0.09 m	0.5 ± 0.11 m	> 0.05
	Benjamin et al., 2018 [34]	0.69 m	0.69 m	> 0.05
Stride Length	Esposito et al., 2020 [32]	1.08 ± 0.17 m	1.15 ± 0.12 m	> 0.05
Stance Time	Bianchi et al., 2020 [30]	1.20 (1–1.2) s	1.20 (1.1–1.3) s	> 0.05
	Benjamin et al., 2018 [34]	0.65 s	0.67 s	> 0.05
Base of Support	Esposito et al., 2020 [32]	0.10 ± 0.03 m	0.10 ± 0.04 m	> 0.05

*MP* Medial pivot, *PS* Posterior stabilised, *SD* Standard deviation, *m* Metres, *s* Seconds, *min* Minutes

### Clinical scores

Seven studies reported clinical and functional scores (Table 6) [27, 29–32, 34, 35]. Three studies [31, 34, 35] utilized the Knee Society Clinical Score (KSCS), and one study [35] among them also analyzed the Knee Functional Score (KFS). Two studies [32, 34] analyzed the Oxford Knee Score (OKS). No statistically significant differences were found between MP and PS TKA patients in terms of these three scores. Two studies [29, 30] utilized the Forgotten Joint Score (FJS), and in one [30] of these studies, a statistically significant difference was noted in favor of the MP TKA. Finally, one study [27] reported the Western Ontario and McMaster Universities Arthritis Index (WOMAC) score, and no statistically significant differences were found in all the subscale results analyzed except for the stiffness, which was found to be significantly higher in MP TKA.

**Table 6:** Clinical scores

**Clinical Scores**	**Author and Publication Year**	**MP TKA**	**PS TKA**	***P* Value**
		**Mean Value ± SD/Range**	**Mean Value ± SD/Range**	
KSCS	Miura et al., 2020 [31]	84.9 ± 23.2	82.9 ± 23	> 0.05
	Benjamin et al., 2018 [34]	85.1 (53–100)	87.2 (55–100)	> 0.05
	Papagiannis et al., 2016 [35]	84.6 ± 5.7	85.5 ± 5.1	> 0.05
KFS	Papagiannis et al., 2016 [35]	83.8 ± 5.01	85.9 ± 5.35	> 0.05
OKS	Esposito et al., 2020 [32]	43.6 ± 3.4	45.4 ± 2.5	> 0.05
	Benjamin et al., 2018 [34]	39.6 (28 to 48)	40.4 (25 to 48)	> 0.05
FJS	Tan et al., 2021 [29]	60.7 ± 15.35	51.3 ± 17.62	> 0.05
	Bianchi et al., 2020 [30]	91.9 (88–95)	75.3 (68–82)	** < 0.05**
WOMAC score, mean total	Stolarczyk et al., 2022 [27]	29.3	24.6	> 0.05
WOMAC score, mean function	Stolarczyk et al., 2022 [27]	22.6	19.6	> 0.05
WOMAC score, mean pain	Stolarczyk et al., 2022 [27]	3.7	3.8	> 0.05
WOMAC score, mean stiffness	Stolarczyk et al., 2022 [27]	3	1.1	** < 0.05**

*MP* Medial pivot, *PS* Posterior stabilised, *SD* Standard deviation, *KSCS* Knee Society Clinical Score, *KFS* Knee Functional Score, *OKS* Oxford Knee Score, *FJS* Forgotten Joint Score, *WOMAC score* Western Ontario and McMaster Universities Arthritis Index score

## Discussion

This study aimed to comprehensively understand kinetic and kinematic differences between MP and PS TKA designs. Particular attention was paid to tibiofemoral movements, spatio-temporal parameters, and clinical scores between patients treated with MP and those with PS TKA. Several significant differences between the two prosthetic designs were observed in this systematic review.

The analysis of kinematic parameters exhibited a higher knee flexion peak in the swing phase, greater knee flexion at toe-off and an increase in the adduction angle in the PS TKA relarive to MP TKA. The kinetic evaluation revealed that the MP TKA designs had a greater maximum flexion and rotation moment, while the PS TKA presented a higher extension moment than MP TKA. The PS TKA design showed an increased anterior femoral roll and anterior translation on medial and lateral condyle during knee flexion, while the MP TKA showed a greater tibia external rotation than PS TKA. No differences were reported between the two prosthetic designs in spatio-temporal parameters. Finally, the clinical evaluation demonstrated that the MP TKA had a better FJS score and a statistically significant stiffness in terms of the WOMAC score compared to the PS TKA.

### Stiff-knee pattern

A typical "stiff knee pattern", characterized by a knee flexion reduction during the initial phase of the swing from toe-off to the peak knee flexion, was observed in both designs. This condition was caused by a compensatory mechanism, known as "quadriceps avoidance", typical of patients with terminal knee osteoarthritis, to limit anterior pain due to quadriceps femoris contraction [39, 40]. Esposito et al*.* demonstrated that the "stiff-knee pattern" was emphasized in MP TKA compared to PS TKA during peak knee flexion or knee flexion at toe-off: the kinetic results presented in this study supported the kinematic data, having a reduction in maximum knee flexion and extension moment, resulting in clinically lower forces at the patella-trochlear junction [32]. In their electromyographic gait analysis evaluation, the same authors observed that muscle activation time of the rectus femoris, biceps femoris and vastus medialis were significantly lower in the MP TKA compared to both the PS TKA and the control group. Therefore, the PS TKA kinematics during gait was more similar to the physiological non-arthritic knees’ kinematics [32]. Comparable data were also observed by Miura et al*.* [31], who reported reduced knee flexion in the healthy control group in both MP and PS TKA without finding statistically significant differences between the two prosthetic designs.

### Paradoxical anterior femoral roll

The "paradoxical anterior femoral roll" during knee flexion was reported in both MP and PS TKA designs. Several studies have described paradoxical anterior femoral sliding and incorrect tibiofemoral axial rotation during knee flexion in PS TKA in relation to healthy knees [41–43]. Compared to other TKA models, MP TKA may limit anterior femoral translation during knee flexion due to the high congruence of the medial compartment [21]. Two of the three studies that analyzed femoral rollback observed that the PS TKA had more statistically significant translation than the MP TKA [29, 33], whereas no difference was reported by Miura et al. in their paper [31]. Contradictory results were reported for the posterior medial condyle translation during knee flexion because. Theoretically, the MP TKA should provide a stable medial compartment, described as a ball and socket, limiting the overall translation. This hypothesis was confirmed by Gray et al*.* in their article [33]. On the other hand, no difference was observed by Tan et al*.* [29]. Two studies reported no statistically significant differences in the lateral femoral condyle anterior translation between the two groups [29, 33].

### Screw-home mechanism

The "screw-home mechanism" represents one of the most critical factors influencing knee stability during standing. It is characterized by relative tibiofemoral axial rotation during the last 20º–30º of knee extension. All studies included in this systematic review reported that MP, as well as PS TKA designs, failed to reproduce this knee mechanism. The screw-home motion is a complex kinematic phenomenon requiring the integrity of anterior cruciate ligament (ACL). The ACL plays a key role as a stabilizer during the late swing/early stance by promoting external rotation of the tibia relative to the femur. Unfortunately, the ACL is currently sacrificed by many PS and MP TKA designs. Among the three studies [29, 31, 33] which analyzed tibiofemoral external rotation during knee flexion, only Gray et al*.* [33] described greater external rotation with the MP TKA design when compared to its PS counterpart. At the same time, many other studies covered by the current review reported no statistically significant differences between the two prosthetic designs [29, 31].

### Limitations

This paper has multiple limitations. First, various manufacturers' MP and PS TKA designs have been analyzed. The articles included in this systematic review examined five different MP and PS TKA models. In addition, the inclusion of both single-radius and multi-radius TKA may result in kinematic changes of knee. In particular, the radius of condylar curvature has a great impact on reducing pressure in the patellofemoral joint, preventing paradoxical anterior motion, and improving quadriceps efficiency. Second, the number of TKAs included in some studies was small, so the systematic review may be under-powered. Third, all gait analysis studies included in the current review were performed using different cameras, force plates, walking surfaces, walking speed and time after surgery. Finally, various clinical and functional scores were analyzed in the included studies. More homogenous use of the implants, a more standardized gait analysis protocol and clinical and functional evaluation might improve data validity.

## Conclusion

This systematic review confirmed that important kinematic and kinetic differences exist between MP and PS TKA designs, but both TKAs kinematics are quite distant from that of a healthy knee. Patients who underwent PS TKA appeared less affected by the stiff-knee pattern. Both prosthetic designs showed an "undesired" paradoxical anterior femoral motion in the early stance phase. This phenomenon was, however, less pronounced in the MP TKA design. Finally, both designs were ultimately unable to reproduce the screw-home mechanism.

## Data Availability

The dataset analysed in this study is available from the corresponding author on reasonable request.
